# Microfluidic-Assisted *Caenorhabditis elegans* Sorting: Current Status and Future Prospects

**DOI:** 10.34133/cbsystems.0011

**Published:** 2023-04-14

**Authors:** Hang Yuan, Wenwen Yuan, Sixuan Duan, Keran Jiao, Quan Zhang, Eng Gee Lim, Min Chen, Chun Zhao, Peng Pan, Xinyu Liu, Pengfei Song

**Affiliations:** ^1^ School of Advanced Technology, Xi'an Jiaotong - Liverpool University, Suzhou, China.; ^2^ Department of Electrical and Electronic Engineering, University of Liverpool, Liverpool, UK.; ^3^ Department of Chemistry, Xi’an Jiaotong-Liverpool University, Suzhou, China.; ^4^ Department of Mechanical & Industrial Engineering, University of Toronto, Toronto, Canada.

## Abstract

*Caenorhabditis elegans* (*C. elegans*) has been a popular model organism for several decades since its first discovery of the huge research potential for modeling human diseases and genetics. Sorting is an important means of providing stage- or age-synchronized worm populations for many worm-based bioassays. However, conventional manual techniques for *C. elegans* sorting are tedious and inefficient, and commercial complex object parametric analyzer and sorter is too expensive and bulky for most laboratories. Recently, the development of lab-on-a-chip (microfluidics) technology has greatly facilitated *C. elegans* studies where large numbers of synchronized worm populations are required and advances of new designs, mechanisms, and automation algorithms. Most previous reviews have focused on the development of microfluidic devices but lacked the summaries and discussion of the biological research demands of *C. elegans*, and are hard to read for worm researchers. We aim to comprehensively review the up-to-date microfluidic-assisted *C. elegans* sorting developments from several angles to suit different background researchers, i.e., biologists and engineers. First, we highlighted the microfluidic *C. elegans* sorting devices' advantages and limitations compared to the conventional commercialized worm sorting tools. Second, to benefit the engineers, we reviewed the current devices from the perspectives of active or passive sorting, sorting strategies, target populations, and sorting criteria. Third, to benefit the biologists, we reviewed the contributions of sorting to biological research. We expect, by providing this comprehensive review, that each researcher from this multidisciplinary community can effectively find the needed information and, in turn, facilitate future research.

## Introduction

*Caenorhabditis elegans* (*C. elegans*) has been a model organism for biochemical and medical research since its discovery by Sydney Brenner in the 1970s, who was awarded the Nobel Prize for recognizing the importance of this discovery in the genetic regulation of organ development and programmed cell death [1–3]. *C. elegans* is a small whole organism; compared to single cell lines and isolated tissues, it offers a much more powerful platform for biological and medical studies by enabling multicellular and multitissue environments [4–6]. Moreover, *C. elegans* is particularly powerful for modeling the nervous system, compared to the other small model organisms, thanks to its high conservation (~65%) of genetic pathways with humans [5,7,8]. This unique advantage provides one excellent platform to study complex human neurodegenerative diseases such as Alzheimer's disease and Parkinson's disease [1,9–12]. In addition, *C. elegans* is the first multicellular model organism to have its entire gene sequenced [7,13–15]. Also, it has high-level behavioral phenotypes such as chemotaxis [16–19], electrotaxis [16,20–28], and learning and memory [29–31], making it a unique value in biology genetics. *C. elegans* has transparent bodies and is ideal for optical in vivo imaging [32–38], making it convenient for neuron fluorescent labeling and imaging [7]. Finally, *C. elegans* is small in size (1 to 1.3 mm), easy to culture, has a short developmental cycle (3 to 4 d) and life cycle (2 to 3 wk) [39], and thereby is inexpensive for large-volume studies [11,12,40,41]. The advantages of *C. elegans* spark many research interests and advances in biology, medicine, and engineering, among others [39,42–44].

**Fig. 1.**
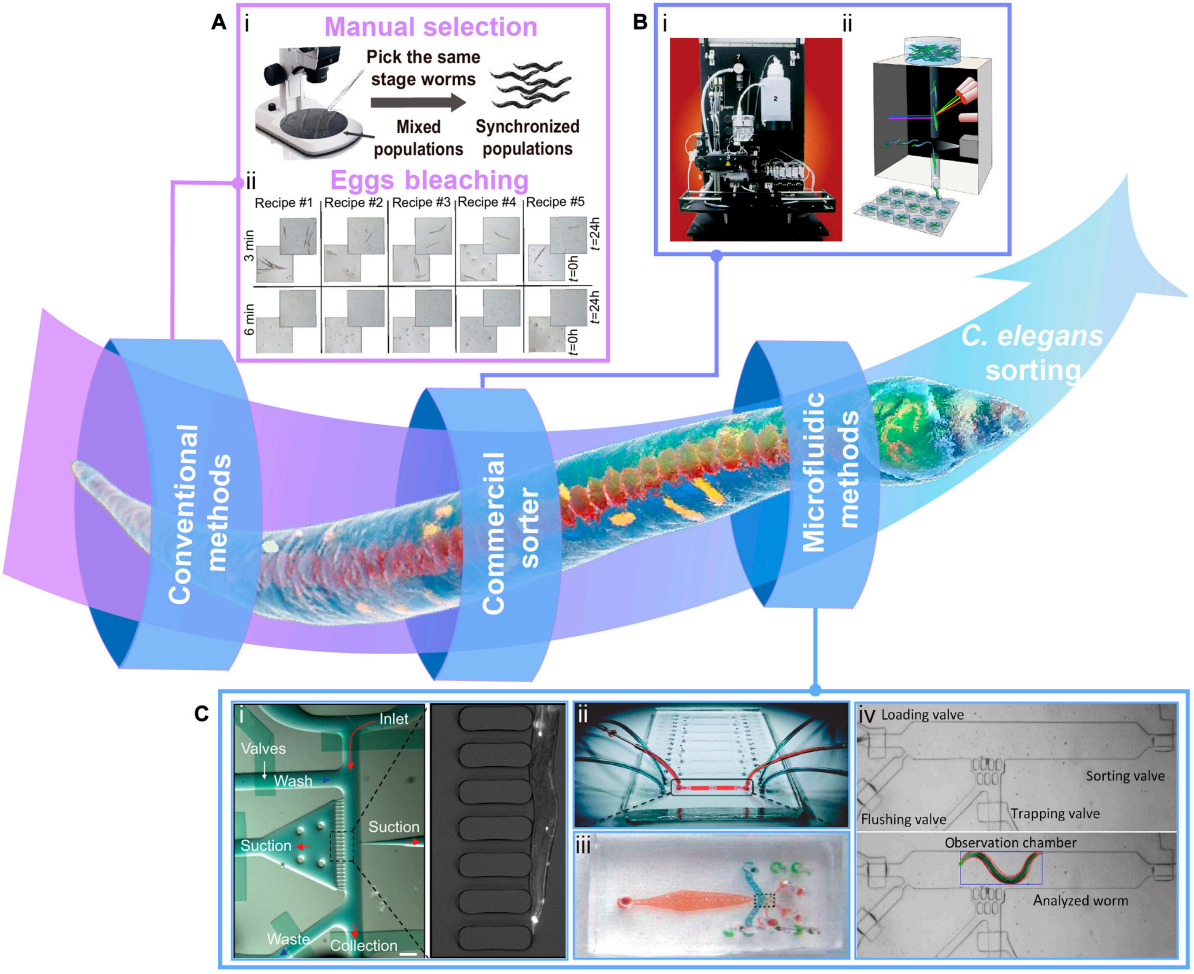
The development of *C. elegans* sorting. (A) Conventional methods. (i) Manual selection. Experimenters selected worms of the same stage from mixed worm populations under a microscope to obtain synchronized populations. (ii) Egg bleaching. Reproduced with permission from [127]. Copyright 2012 *Journal of Visualized Experiments*. (B) Commercial sorter. (i) Internal view of the COPAS system. Reproduced with permission from [58]. Copyright 2006 Springer Nature. (ii) The COPAS Biosorter is used for worm sorting. Reproduced with permission from [128]. Copyright 2018 Springer Nature. (C) Microfluidic methods. (i) Photograph of a microfluidic on-chip sorter and a single worm trapped by multiple suction channels. Reproduced with permission from [73]. Copyright 2007 National Academy of Sciences, U.S.A. (ii) Photograph of the entire microchip with 8 individual worm selection units (1 with tubes connected). Reproduced with permission from [49]. Copyright 2016 Royal Society of Chemistry. (iii) Photograph of the microdevice. Reproduced with permission from [50]. Copyright 2008 Springer Nature. (iv) Photograph of the target worm in the observation chamber of the microfluidic chip. The worm is sorted by measuring the size after loading through the valve. Reproduced with permission from [61]. Copyright 2019 IEEE.

*C. elegans* has 4 distinct larval stages (L1 to L4) and 1 adult developmental stage [39,45], where the *C. elegans* exhibits different biological characteristics, phenotypes (e.g., length, locomotion, and electrotaxis behavior), and behaviors. Most *C. elegans* studies require the worms from 1 specific developmental stage to avoid age-induced heterogeneous phenotypic effects [46]. Therefore, *C. elegans* sorting has been an essential task for work research communities. Usually, worm length can be used as one indicator to classify the developmental stages of selected worms [46–49]. Further, fluorescence-based microfluidic devices (e.g., the difference in optical signal when worms pass through optical fibers) are more widely used to sort mutants from the wild-type *C. elegans* [50]. To obtain the stage-synchronized worm populations, one conventional method is the manual selection of the specific worms from the mixed populations under the microscope (Fig. 1A) [22,36,41,49–56]. Synchronizing large numbers of worms in this way is time-consuming, labor-intensive, and low-throughput.

The worms can also be synchronized by hypochlorite bleaching the gestational hermaphrodite worms to harvest the embryos and are preselected by handpicking to maintain the embryos in identical stages (Fig. 1A). The cultured worms are then in the same stage. This simultaneous cultivation method is criticized for the drawbacks of time consumption and low productivity and also requires high operational skills. The whole process is cumbersome and invasive [48,57].

Later, the commercial complex object parametric analyzer and sorter (COPAS) was developed to high-throughput sort the worms on the basis of fluorescent signals (protein expression) and other optical features (Fig. 1B) [58,59]. This technology, similar to flow cytometry, rapidly measures important biophysical and biochemical characteristic covariates that can sort specified cell subpopulations on the basis of a preselected covariate range [60]. It is high-efficient, but its measurement algorithm is fixed and cannot be customized according to specific experimental requirements, which hinders its wide use in *C. elegans* research [61]. More importantly, these sorters are bulky and expensive, limiting the operating environment in which they can be used and increasing the financial burden. Therefore, worm laboratories still desire easy- and fast- sorting devices to operate with high throughput on large datasets at a reasonable cost.

Most recently, microfluidics has emerged as one promising platform that advances *C. elegans* research by enabling sorting (Fig. 1C), injecting, drug screening, and many other studies, thanks to its perfect size match (in the range of hundreds of microns to a few millimeters) [62–66]. Also, most microfluidic platforms are made of silicone elastomer polydimethylsiloxane (PDMS) [67–71], which is biocompatible and air-permeable [7,72], and thereby suitable for on-chip studies of the entire worm life cycle. Meanwhile, microfluidics can be integrated with on-chip micropumps, microvalves, and automated controllers to automate the flow process [50,73,74]. Microfluidic platforms can also be connected to external hardware such as temperature, pressure, and red-green-blue sensors for data feedback and control, enabling intelligent systems. In addition, microfluidic devices are miniaturized to allow for higher sample densities and throughput. This feature makes microfluidic devices suitable for various experimental operations for this tiny worm, such as sorting, screening, optical imaging, and microinjection [75–78]. Therefore, microfluidic devices, including the benefits mentioned above, make it possible to quickly and accurately separate worms. Although there are some limitations in the integrated, high-content, and multi-index sorting (e.g., multi-index sorting is hardly integrated on a single microfluidic device), it still provides more laboratories with affordable, small footprints and convenient operation equipment [79].

Most previous reviews are organized from the perspectives of microfluidic engineers, focusing on the development of microfluidic devices, but have not provided summaries and discussion from the perspectives of *C. elegans* research requirements by biologists. In addition, these reviews tried to cover all the microfluidic applications in worm research, including high-throughput screening [7,11,31], manipulation [15,41], and imaging [35,80,81], and a much-focused review, i.e., focusing on the microfluidic *C. elegans* sorting, can be a much more efficient and time-saving tool for researchers. Moreover, many microfluidic devices have been developed for *C. elegans* sorting in recent years that have not been included in previous reviews, calling for an up-to-date review on that aspect.

In this review, we comprehensively reviewed up-to-date advances in microfluidic-assisted *C. elegans* sorting from several angles. First, we described conventional methods for *C. elegans* culturing, transferring, and sorting in Conventional Methods for *C. elegans* Culturing, Transferring, and Sorting and commercial automatic *C. elegans* sorters in Commercial Automatic *C. elegans* Sorters. We highlighted the advantages and limitations of microfluidic-assisted *C. elegans* sorting devices in Comparison of the Current *C. elegans* Sorting Methods. Second, to benefit engineers, we reviewed existing devices from several perspectives, including active or passive sorting in Active and Passive Microfluidic *C. elegans* Sorting Devices, physiological properties in *C. elegans* Physiological Properties Available for Microfluidic Sorting Devices, and sorting strategies in Different Strategies of Microfluidic *C. elegans* Sorting. Third, to benefit biologists, we reviewed the contribution of the technology to biological research in terms of target populations in Different Target *C. elegans* Sorting and sorting criteria in Microfluidic Devices Based on Various Sorting Criteria. Finally, we provided future prospects on biochemical and medical applications of *C. elegans* sorting and the development of sorting methods and devices in Future Prospects for Microfluidic *C. elegans* Sorting Devices. We expected to provide a rapid and effective documentary for the cross-disciplinary field, which can help relevant researchers find the needed information and, in turn, facilitate the research advance in the combination of microfluidics and *C. elegans*.

## Conventional Methods for *C. elegans* Culturing, Transferring, and Sorting

*C. elegans* sorting is essential to keep all the worms at the same developmental stage to produce convincing and accurate data in worm-based assays. The *C. elegans* sorting technique has gone through years of development, including the refinement of methodologies, the establishment of new systems, and the invention of new devices, and various methods of worm sorting have been developed and utilized. Furthermore, it starts with repetitive, tedious, and labor-intensive manual operations, which are gradually replaced by automated tools. This section focuses on the conventional methods for *C. elegans* culturing, transferring, and sorting.

For worm culturing, the temperature is a critical factor influencing *C. elegans* development [9,46,82]. *C. elegans* is usually cultured at temperatures ranging from 16 to 25 °C, with the optimal temperature at 20 °C. Different culture temperatures lead to different worm growth rates, affecting the growth cycle. For instance, the growth rate of *C. elegans* is 2.1 times higher at 25 °C than at 16 °C and 1.3 times higher at 20 °C than at 16 °C [57,83,84]. Therefore, different culturing temperatures may contribute to different diameters and lengths of worms, though worms are in the same developmental stage, which hinders the accurate sorting (age-synchronized) of *C. elegans*. The researcher needs to consider environmental parameters, such as temperature and humidity. In addition, sufficient food, usually the *E. coli* Op50 strain in the laboratory environment, should be guaranteed to prevent the late development of worms due to starvation [3,57,85].

During the past decades, conventional approaches to manual worm culture, transfer, and sorting have been well established. The easiest way to transfer worm populations is by “block transfer”, using a sterilized scalpel or small spatula to cut a large agar block from the old disk and put it on the new one [57]. The worms in the agar block will move onto the moss of the new dish. Another method is to use sterilized filter paper to transfer the worms attached to the filter paper covering the petri dish. After transfer, *C. elegans* are usually fixed by glue or anesthetic for examination under the microscope, and the researchers can pick up the desired worms using a worm pick.

Manual sorting can be performed for size-based worm sorting to obtain age-synchronized worms. The traditional method is to synchronize population ages by bleaching embryos with hypochlorite or by regularly allowing worms to lay eggs on dishes [57]. This is a cumbersome procedure and hypochlorite is highly invasive and may damage the eggshells [48].

Although such conventional methods are relatively well established, there are still common problems that cannot be eliminated, such as being time-consuming, cumbersome, and low-throughput. Manual operation is a low-throughput process where only a single output parameter can be monitored per experiment and does not allow real-time monitoring of worm conditions [7,41]. Moreover, excessive experimental error may affect accuracy and sensitivity (and possibly even harm the worms in the selection), and these methods require trained laboratory personnel to operate and consume large amounts of reagents, time, and labor [57]. For worm sorting based on fluorescent signals or any other subtle indicators, conventional methods are difficult to perform well. Commercial automatic sorting machines have been developed to improve this situation and meet the increasing demand for high-throughput sorting.

## Commercial Automatic *C. elegans* Sorters

**Fig. 2.**
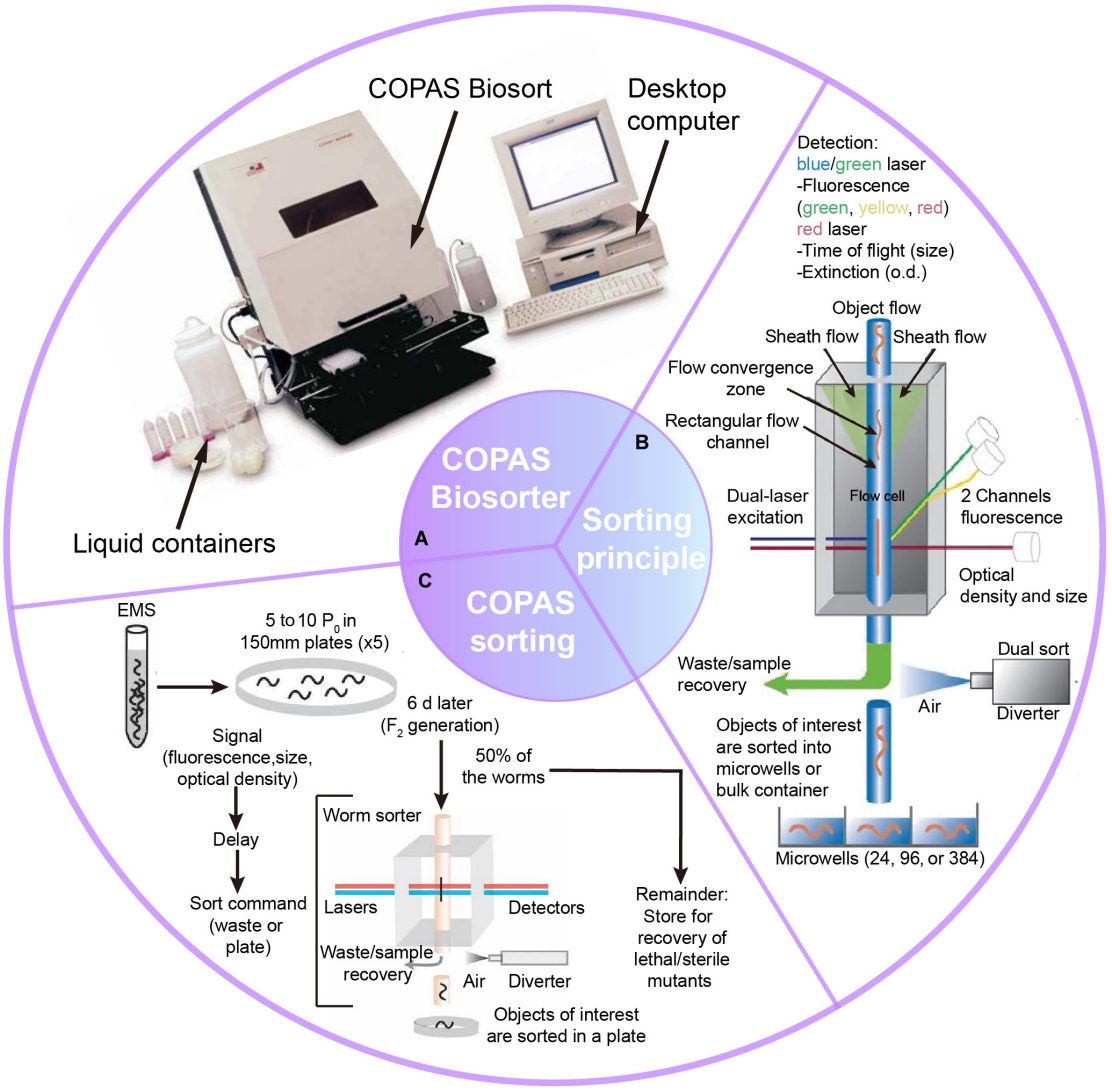
Commercial automatic *C. elegans* sorters. (A) Photograph of the commercial automatic cytometer COPAS Biosorter with the accompanying desktop computer and associated liquid containers and (B) the schematic diagram of the sorting principle. Reproduced with permission from [58]. Copyright 2006 Springer Nature. (C) The process of automated COPAS system sorting for *C. elegans*. Reproduced with permission from [59]. Copyright 2008 Springer Nature. o.d., optical density; EMS, ethyl methane sulfonate.

The demand for reliable, high-throughput sorting systems for *C. elegans* experiments has increased as the complexity and scale of worm experiments increased [86]. Subsequently, the commercially available flow cytometer COPAS Biosorter (Fig. 2A) from Union Biometrica was developed in the late 1990s. It is designed for high-speed, automated analysis and sorting of numerous tiny model organisms, for example, *C. elegans* and zebrafish, among others. As shown in Fig. 2B, the COPAS system is based on the principle of optical analysis using animal length, optical density, fluorescence intensity, and other optical features to analyze and sort live multicellular organisms. Each worm passes through the sensor, and its axial length, extinction (optical density measurement), and fluorescence excitation are measured [58,59]. After analysis, the *C. elegans* that meet user-defined criteria can be dispensed into bulk containers or multiwell plates for high-throughput sorting (Fig. 2C) [58,59,87].

Compared to the conventional manual sorting methods, the COPAS system can achieve higher accuracy than manual operation and with much higher sorting speed and throughput, greatly reducing the experiment time. In addition, manual sorting is limited by the operators' skills, and some human error may occur to damage the worms. The sorting mechanism used by COPAS is pneumatic, gentle, and does not harm the worm itself. In contrast to conventional flow cytometers, the COPAS increases the width of the processed object to 150 to 200 μm through large-bore fluidics and flow cell design [58], and thereby small organisms such as *C. elegans* can be accurately analyzed, sorted, and dispensed at the speed of hundreds per minute. COPAS can continuously perform the sorting and collect relevant data such as length for sorting large numbers of specific populations. It can also be used to separate particular individuals or populations optically different on the basis of fluorescence from vast amounts of worms.

Although powerful, such a machine is expensive, and many laboratories cannot afford it. Moreover, it is bulky, not easy to move, and complex to operate, requiring well-trained personnel. Therefore, for routine worm population sorting, portable, low-cost, miniaturized, easy-to-operate, high-throughput, and high-accuracy microfluidic sorting devices are highly desired.

## Microfluidic Sorting of *C. elegans* and Developmental Stages

### Comparison of the Current *C. elegans* Sorting Methods

Conventional methods are only capable of a single monitored parameter and may damage worms even by well-trained experimenters, and need to face low throughput and precision and high reagent consumption. Although commercial sorters have improved greatly in throughput, accuracy, and automation, the challenges of being expensive, bulky, and highly demanding to operate remain. Microfluidic methods are capable of manipulating and detecting biological samples, reagents, or biomolecules in a microscale environment that can be well matched to the demands of *C. elegans* sorting. Compared with conventional methods and commercial sorters, microfluidic methods have different degrees of improvement, mainly in terms of functionality, manipulation, sensitivity and precision, throughput, cost, and personnel, as shown in Table 1. Over the last 2 decades, the demand for high throughput and the advances in miniaturized analytical techniques, highly accurate sorting, and high automation fashion have accelerated microfluidics’ usage in *C. elegans* sorting techniques [88,89].

**Table 1.** The comparison of the current *C. elegans* sorting methods.

	Conventional method	Commercial sorters	Microfluidic method
Functionality	Single monitored parameter	Multiple monitored parameters	Multiple monitored parameters
Manipulation	Manual with human error and worm-damaged risk	Automatic and gentle	Semi- or fully automated and gentle
Sensitivity and precision	Low sensitivity and imprecise external stimuli	High sensitivity, high resolution, and precision	High sensitivity and a precise small amount of external stimuli
Throughput	Low	High	High
Cost	Expensive (large reagent consumption)	Expensive (bulky and costly equipment)	Cost-effective, miniaturized, portable, and disposable
Personnel	Well-trained and skilled operators	Well-trained and skilled operators	Minimal personal training requirements, user-friendliness

*C. elegans* research greatly benefited from microfluidic devices and is the perfect match. First, the sizes of microchannels and *C. elegans* are in similar ranges (hundreds of microns). Second, thanks to the microstructure, high-throughput (e.g., hundreds of worms per minute) operations are possible at a single animal resolution. Third, when combined with automation technologies, high-speed detection, high-content, and high-sensitivity analysis can be achieved. Fourth, when combined with microscopic imaging algorithms, real-time monitoring of multiple parameters for high-content sorting is also viable. Fifth, microfluidic devices are usually cost-effective and disposable. Sixth, automated microfluidic systems can be developed with the user-friendly interface, enabling minimal training requirements for operators. Otherwise, thanks to low-cost polymer manufacturing technologies like soft lithography [72], multiple complicated, disposable, biocompatible structures for rapid testing and development can be fabricated. Therefore, the research communities have quickly adapted and employed microfluidic devices based on *C. elegans* sorting methods.

### Active and Passive Microfluidic *C. elegans* Sorting Devices

One of the most straightforward ways to classify microfluidic sorting devices into active and passive categories is based on whether or not *C. elegans* has been sorted by active forces. Table 2 provides an overview of these active and passive devices and their corresponding advantages.

**Table 2.** An overview of active and passive sorting devices and main advantages.

Device feature(s)	Active or passive	Advantage(s)	Reference
Single-layer PDMS			
Arrays of geometrically optimized square columns	Active	Directed sorting with high efficiency (>96%), accuracy (>95%), and throughput (∼120 worms/min) and screening for size mutants	[48]
Optical fiber detection and laminar flow switching	Active	High throughput (60 worms/min), sorting accuracy (96.6%), and switching accuracy, gentle for worms	[112]
Arrays of chambers and clamps	Active	Longitudinal measurement of size and locomotion	[131]
Partially closed valves	Active	Highly reliable quantitative adaptive algorithm for high-throughput (~83.33 worms/min) sorting	[92]
A serpentine channel and an array of circular chambers	Passive	Parallelization, without costly and active off-chip components, longitudinal behavioral tracking	[132]
Local electric field traps and semicontinuous flow	Passive	Effective and automatic sorting using electrotaxis based on locomotion first (78 worms/min)	[20]
The electric field, hexagonally arrayed microstructures	Passive	Maximize worm motility for directed self-sorting under the electric field	[22]
Visual aid and electrophoresis box	Passive	Simple and quantitative measurement for self-sorting	[24]
Microchannels with electrodes	Passive	Cost-effective and sensitive sorting using electrotaxis	[25]
Geometrically optimized pillars	Passive	High-throughput (129 ± 31 worms/min) and purity (~96.8%) sorting with an average efficiency of 95%	[51]
Interconnecting channels (Smart mazes)	Passive	Repeated passive sorting to avoid clogging and efficiently separate adult and larval worms	[54]
Special angled symmetrical channels with electric field	Passive	Efficient, economic, and harmless stage-specific deflecting electrotactic responses sorting	[55]
Spiral channels with a trapezoidal cross-section	Passive	High-throughput (4200 worms/min) and high-accuracy (>95%) sorting without chemical	[93]
Microchannels applied dc and ac electric fields	Passive	Effective worm immobilization and high-throughput automated analysis	[133]
Inclined surface with conduit	Passive	High-throughput sorting using the surface following	[124]
Two-layer PDMS			
Vision-assisted sorting valves	Active	High-throughput (>3.67 worms/min) quantitative phenotypic sorting of mutants	[134]
Pneumatic microvalves, micropillars, and microelectrodes	Active	Individual worms can be nondestructively recovered after electrophysiological phenotype-based sorting	[74]
Pneumatic microvalves and distributary channels	Active	High-throughput (30 worms/min) sorting based on label-free electrical impedance spectroscopy	[110]
Algorithm-assisted valves	Active	Anticlogging, gentle, high-accuracy (>95%) and high-throughput (15 worms/min) sorting	[50]
Algorithm-assisted, pressure-controlled valves	Active	Robust, real-time worm size measurement and high-throughput (∼10.34 worms/min) sorting	[61]
Circular arranged micropillars and multiple control valves	Active	High-speed (<100 ms per frame) image acquisition for sorting, anticlogging flow at high concentrations	[73]
Specific shape diode arrays	Passive	Directed high-throughput (97±4 worms/min) sorting	[47]
Adjustable filter structures	Passive	High-efficiency (~100%), high-purity (~100%), and high-throughput (210 worms/min) sorting	[49]
Curved channels and valves	Passive	Lateral positioning and efficient (85%) mutant sorting	[135]
Multiple-layer PDMS			
Aspiration channels	Active	Reusable, stable, and noninvasive fixation for sorting	[56]
Adjustable filter structures	Passive	Reusable, high-purity (73%–100%) and high-throughput (160–240 worms/min) sorting and eggs extraction	[46]

**Fig. 3.**
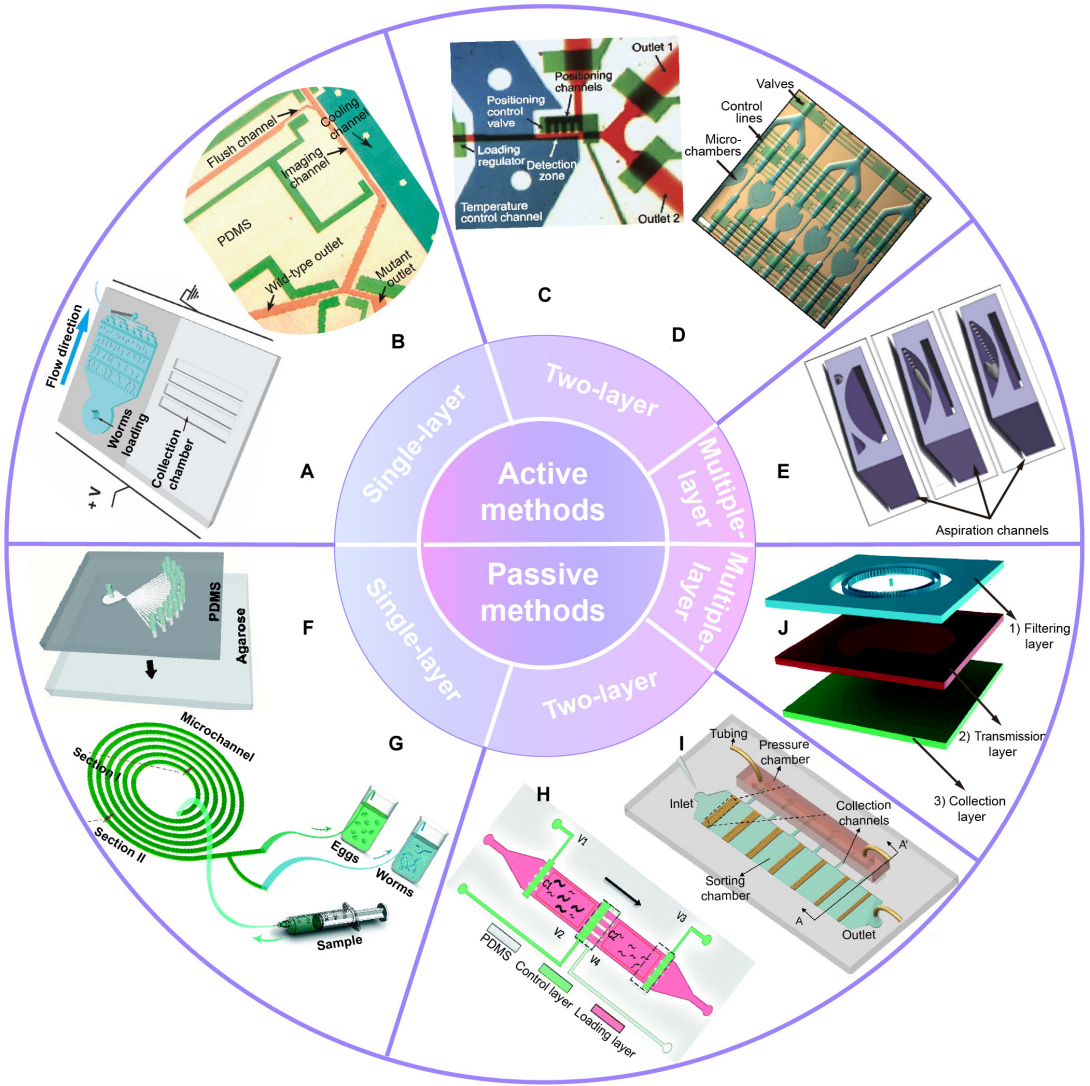
Active and passive sorting on-chip devices. (A) Single-layer PDMS-agarose hybrid microfluidic graduated microfilter device. Reproduced with permission from [48]. Copyright 2018 Elsevier. (B) Single-layer PDMS device with cooling channels (blue), control valves (green), and sample flow channel (red). Reproduced with permission from [92]. Copyright 2012 Oxford University Press. (C) Two-layer PDMS device with on-chip valves. Reproduced with permission from [50]. Copyright 2008 Springer Nature. (D) Two-layer PDMS device with valves, multiplexed control lines, and microchambers. Reproduced with permission from [73]. Copyright 2007 National Academy of Sciences, U.S.A. (E) Multiple-layer PDMS device with aspiration channels. Reproduced with permission from [56]. Copyright 2008 Royal Society of Chemistry. (F) Single-layer PDMS-agarose device with special angled symmetrical sorting channels and electric field. Reproduced with permission from [55]. Copyright 2015 Royal Society of Chemistry. (G) Single-layer PDMS device with spiral microchannels. Reproduced with permission from [93]. Copyright 2018 Royal Society of Chemistry. (H) Two-layer PDMS device with adjustable filter structures. Reproduced with permission from [49]. Copyright 2016 Royal Society of Chemistry. (I) Two-layer PDMS device with diode arrays. Reproduced with permission from [47]. Copyright 2017 Springer Nature. (J) Multiple-layer PDMS device with filter structures. Reproduced with permission from [46]. Copyright 2020 Royal Society of Chemistry.

Active sorting methods are mainly based on the active forces to alter the targeted *C. elegans* to the defined outlets and thereby achieve sorting purposes. The microvalves [50,73] and micropumps are usually used, allowing worm populations that match sorting criteria to be sorted out by flow and pressure. The droplet-based method can also be categorized as one of the active methods, as the valve is normally needed to alter the droplet flows. In particular, droplets usually enable high-throughput sorting [32,90,91]. Wang et al. combined an electric field with flow drive to use electrotaxis for the rapid directional passage of worms through microfilters. The device can also screen mutants with abnormal sizes and retrieve classified worms (Fig. 3A) [48]. Active sorting methods are usually performed with the aid of collected images or other signal feedback. Lee et al. [92] fabricated the imaging, loading, flush, and cooling channels in single-layer PDMS for rapid high-throughput screening (Fig. 3B). Similarly, the method of active sorting based on phenotypic imaging was also used in the microfluidic platform of Chung et al. (Fig. 3C) [50]. This platform only has one single imaging channel where the individual worms can be immobilized by cooling the temperature and controlling the valves. Rohde et al. [73] also developed a microfluidic device with circularly arranged micropillars and multiple control valves that can be used for both high-throughput phenotypic sorting and rapid chemical screening (Fig. 3D). *C. elegans* with abnormal phenotypes that are identified by imaging are sorted by the fluid flow directly to the waste outlet. Zeng et al. [56] fabricated multiple-layer PDMS devices with aspiration channels that harmlessly reduce the mobility of worms, and thereby high-resolution fluorescent imaging can be achieved (Fig. 3E). This facilitates the further step to perform several possible manipulations, including sorting. Dong et al. [61] also developed a microfluidic device with computer-controlled pneumatic valves for sequential loading, capturing, measuring, and size-based sorting of single worms. The vision-based and algorithm-assisted microfluidic system provides accurate morphometry and active real-time sorting to load multiple worms and detect size failure.

Passive sorting devices usually employ specific microstructures such as “micro-bumps” channels [22], micropillars [51], “smart mazes” [54], and spiral microchannels [93] to sort by defining the passable size of the *C. elegans*. The “smart maze” is a typical design of channel structure. The structure consists of isolated micropillars and chambers or “pools” that allow adults and larvae to swim through respectively in channels suitable for their body sizes [54]. Geometric optimization of passive sorting structures such as micropillars, channels, and diodes is usually needed to achieve high sorting efficiency. Ai et al. [51] optimized the chamber height and pillar spacing to optimize the size filtration capacity of the microfluidic device. Wang et al. [55] not only designed symmetrical channels with specific angles on the chip structure but also used deflecting electrotaxis to sort worms’ different developmental stages simultaneously (Fig. 3F). They also found that the locomotion of *C. elegans* was proportional to the electric field intensity.

Several special microstructured passive sorting microfluidic devices also overcome some challenges of conventional filtration techniques, such as mechanical damage, nonreproducibility, and clogging. Sofela et al. [93] designed a microfluidic device with spiral microchannels to separate *C. elegans* eggs utilizing the inertial lift and drag forces (Fig. 3G). This microstructure eliminates the clogging problem. Dong et al. [49] also developed a microfluidic device with adjustable filter structures, which is achieved by the pressure-controlled effective cross-section of microchannels (Fig. 3H). The device reduces the clogging that may result from particles in the filtrate, especially very sticky bacteria clusters, and allows continuous sorting of specific stages of worms. A microfluidic diode structure was designed by Yang et al. [47], in which worms entered from the curved end but avoided swimming out from the straight side (Fig. 3I). This can prevent clogging that might be caused by the presence of eggs and debris when sorting mixed populations. Thus, the sorting efficiency and accuracy at low flow rates were improved. In addition to optimizing the geometrical parameters of the structure, the combination of different filters can also be used to achieve mixed population purification and fine sorting. By combining multiple filter structures with adjustable pore sizes, precise sorting of any 2 continuous worm developmental stages can be achieved. Furthermore, this microfluidic device is reusable and nonclogging (Fig. 3J) [46].

### *C. elegans* Physiological Properties Available for Microfluidic Sorting Devices

There is an approach that utilizes *C. elegans*' inherent physiological properties to passively influence the *C. elegans* locomotion behavior. The implementation of this approach is based on the ability of *C. elegans* to exhibit specific behavioral responses to external physical and chemical stimuli. For instance, different ages, sizes, and phenotypes of worms exhibit different locomotion behaviors toward the local electric fields, and thereby by properly designing the microfluidic devices, the specific type of worms can be sorted out [20–28,94–99]. External stimuli and their corresponding behavioral responses are synthesized in Table 3.

**Table 3.** Summary of external stimuli to *C. elegans* and its corresponding behavioral responses.

Physiological properties	External stimuli	Methods of applying the stimulus	Behavioral responses	Reference(s)
Electrotaxis	Electric field	Connect the voltage source or insert the electrodes	Follow the electric field direction	[20–28,94–99]
Chemotaxis	Chemicals (water-soluble substances)	Specific-channel inner diameter and structure	Forage feed or avoid harmful substances	[17,18,37,85,100,125,136–141]
Rheotaxis	Stream	Syringe pump	Upstream movement	[101–103]
Phototaxis	Light (blue)	LED light	Light avoidance or muscle contraction	[104–106,142–146]
Thermotaxis	Temperature (*T*_c_)	The flow of different temperatures	Avoid the T_c_ on the thermal gradient	[107,147–154]
Aerotaxis	Oxygen %	Inject oxygen (air) via a syringe pump, or PDMS free diffusion	Keep away from the oxygen %	[108,109,155]

**Fig. 4.**
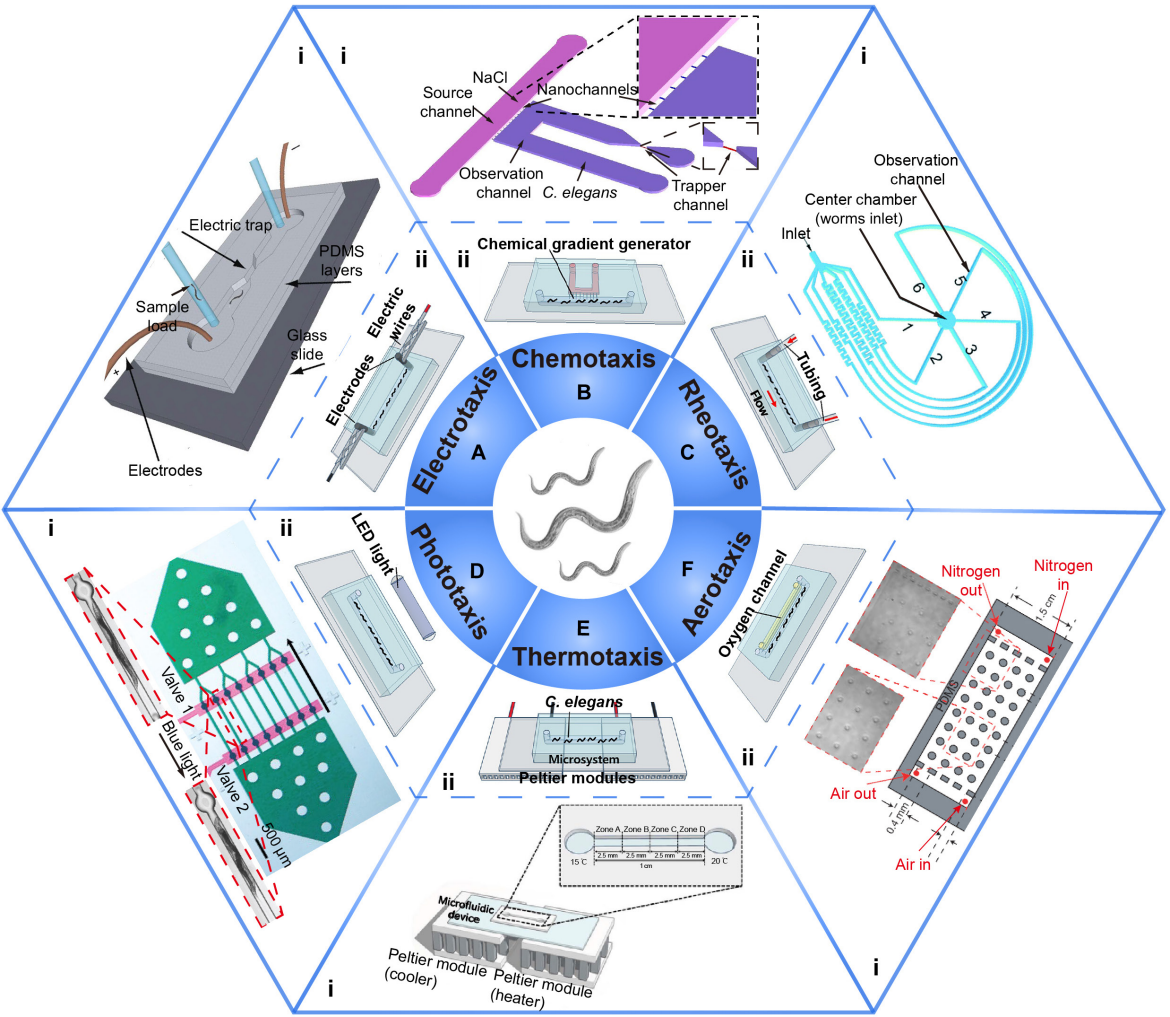
*C. elegans* physiological properties available for microfluidic devices and schematic figures of the devices. (*C. elegans* [center figure]. Reproduced with permission from [129]. Copyright 2016 John Wiley and Sons.) (A) Microfluidic device applying *C. elegans* electrotaxis. (i) Microfluidic device for worm electrotactic sorting (single electric trap). Reproduced with permission from [20]. Copyright 2012 Royal Society of Chemistry. (ii) Schematic figure of the *C. elegans* electrotaxis microsystem. Reproduced with permission from [130]. Copyright 2021 Elsevier. (B) Microfluidic device applying *C. elegans* chemotaxis. (i) Micro/nanofluidic device for *C. elegans* chemotaxis experiment. In parallel microchannels, *C. elegans* showed different chemotaxis according to the different NaCl concentrations. Reproduced with permission from [100]. Copyright 2014 Springer Nature. (ii) Schematic figure of the *C. elegans* chemotaxis microsystem. Reproduced with permission from [130]. Copyright 2021 Elsevier. (C) Microfluidic device applying *C. elegans* rheotaxis. (i) Microfluidic device for the analysis of *C. elegans* rheotaxis consists of an inlet, a flow velocity generator, and a central chamber for the injection of *C. elegans*. Loaded *C. elegans* entered the observation microchannel, chose their preferred flow velocity, and swam upstream. Reproduced with permission from [102]. Copyright 2018 Royal Society of Chemistry. (ii) Schematic figure of the *C. elegans* rheotaxis microsystem. Reproduced with permission from [130]. Copyright 2021 Elsevier. (D) Microfluidic device applying *C. elegans* phototaxis. (i) Microfluidic device for investigating *C. elegans* phototaxis, which consists of 8 parallel trapping channels. After the blue-light illumination to the *C. elegans*, the worms showed contraction (red box). Reproduced with permission from [106]. Copyright 2010 Elsevier. (ii) Schematic figure of the *C. elegans* phototaxis micro-system. Reproduced with permission from [130]. Copyright 2021 Elsevier. (E) Microfluidic device applying *C. elegans* thermotaxis. (i) Microfluidic device with the Peltier module for generating the linear temperature (temperature gradients) in a microchannel. *C. elegans* showed different thermotaxis according to the different temperature ranges. Reproduced with permission from [107]. Copyright 2017 Springer Nature. (ii) Schematic figure of the *C. elegans* thermotaxis microsystem. Reproduced with permission from [130]. Copyright 2021 Elsevier. (F) Microfluidic device applying *C. elegans* aerotaxis. (i) Gas-phase PDMS microfluidic device for generating the oxygen gradient by injecting air and nitrogen gas. The *C. elegans* aggregated and moved toward specific oxygen concentration areas. Reproduced with permission from [109]. Copyright 2004 Springer Nature. (ii) Schematic figure of the *C. elegans* aerotaxis microsystem. Reproduced with permission from [130]. Copyright 2021 Elsevier. LED, light-emitting diode.

*C. elegans* exhibits a directional behavioral response called “electrotaxis” under an electric field gradient. This behavioral response can be applied to sorting on the basis of stage, size, or response level. Rezai et al. [20] developed the first microfluidic system for the electrotaxis behavior of *C. elegans*. Different stages of *C. elegans* exhibited different speeds and response levels in the local electric field traps and were sorted in a semicontinuous flow (Fig. 4A). Compared to conventional agar media, microfluidic platforms are more powerful for controlling chemical concentrations or gradients and for precise delivery at small chemical doses, facilitating chemotaxis studies. Wang et al. [100] developed a micro/nanofluidic device that controlled parallel nanochannel size to generate and adjust chemical concentration gradients (Fig. 4B). According to the stimulation of different NaCl concentrations, *C. elegans* showed different chemotaxis and were sorted. Similarly, syringes, tubes, and pumps can easily connect to channels in microfluidic systems, creating flow conditions for rheotaxis studies [101–103]. A microfluidic device with 6 spiral microchannels that generate different flow velocities based on differences in channel length and shape was designed by Ge et al. [102] (Fig. 4C). Utilizing flow rate differences, this device can screen mutants on the basis of the rheotaxis of different genotypes of *C. elegans*. *C. elegans* exhibits phototaxis (avoid blue light) like those animals with photosensitive organs, though it does not have light-sensitive organs like eyes [104,105]. Stirman et al. [106] designed a microfluidic device with 8 parallel trapping channels (Fig. 4D). With blue-light illumination, *C. elegans* exhibited significant muscle contraction in the microchannels and was easily imaged for further sorting. The thermotaxis of *C. elegans* depends mainly on the culture temperature gradient. The microfluidic system developed by Yoon et al. [107], which consists of a linear microchannel and Peltier modules, can precisely control temperature gradients (Fig. 4E). Wild-type and mutant worms exhibited different thermotaxis according to the different temperature ranges, allowing the device for mutant screening. The behavioral responses of *C. elegans* that adapt to environmental oxygen levels are also significant, and aerotaxis is utilized to change locomotion speed and steer behavior [108]. Gray et al. [109] designed a gas-phase PDMS microfluidic device for generating the oxygen gradient by injecting air and nitrogen gas (Fig. 4F). *C. elegans* aggregated and moved toward specific oxygen concentration areas.

### Different Strategies of Microfluidic *C. elegans* Sorting

Sorting strategies are the main basis for microfluidic *C. elegans* sorting devices, depending on different working principles. For example, *C. elegans* microfluidic impedance cytometry (CeMIC) and fluorescence-assisted sorting are widely used for age synchronization, drug screening, and more. Table 4 summarizes the main strategies of microfluidic *C. elegans* sorting and their principles.

**Table 4.** The main strategies of microfluidic *C. elegans* sorting and their principles.

Sorting strategy	Principle	Applications	Reference(s)
Stimulus response-based	*C. elegans* have behavioral responses to external stimuli (e.g., current pulse, temperature)	Age synchronization, motion-based behavior analysis, drug screening, high-throughput search of biomolecules	[20–22,24,25,28,48,55,96,97,100,102,107,130,133]
CeMIC	Measure electrical impedance during worm flow and identify developmental stages by signals	Age synchronization, drug-evaluation studies, label-free quantification, phenotyping	[110,156]
Pressure-based	Control channel pressure through micropumps, microvalves, or adjustable filter structures	Age synchronization, culturing, stimulation, phenotyping, microsurgery	[49,51,56,61,73,74,92,135]
Continuous flow-based	Fill or perfuse the channel with a single fluid (e.g., culture medium aqueous solution)	Age synchronization, culturing, stimulation, high-throughput imaging	[46–48,54,93,111,112,122,131,132]
Droplet-based	Generate microdroplets (commonly aqueous droplets surrounded by oil) to wrap *C. elegans* for encapsulation	Individual separation, long-term behavioral observation studies, drug screening, high-resolution imaging	[32,90,91,157–159]
Fluorescence-assisted	Detect *C. elegans* fluorescent protein expression and other optical features	High-resolution imaging, genotype analysis, drug screening, biosensing development	[50,112,120,134,160,161]

CeMIC, *C. elegans* microfluidic impedance cytometry

**Fig. 5.**
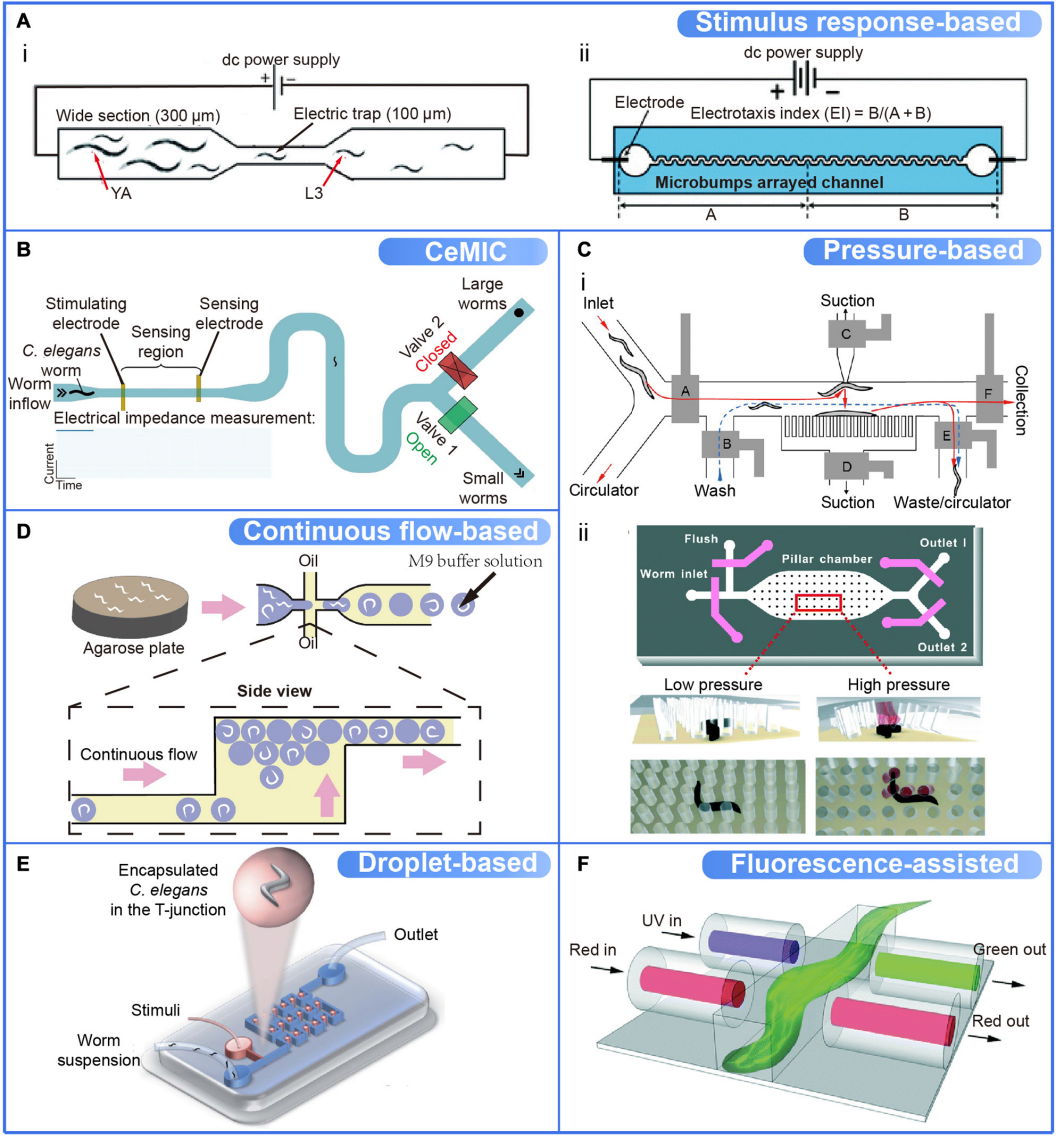
The strategies of microfluidic *C. elegans* sorting. (A) Stimulus response-based microfluidic *C. elegans* sorting devices (eletrotaxis as the example). (i) Single electric trap by narrowing the channel at the center for local electric field enhancement (channel widths mentioned in the parentheses). Reproduced with permission from [20]. Copyright 2012 Royal Society of Chemistry. (ii) The experimental setup for electrotaxis where the PDMS microbumps are arranged in an array in a 2-dimensional hexagonal lattice pattern. Under the induction of electric fields, different size worms exhibit different motility and move orientationally. Reproduced with permission from [22]. Copyright 2012 Royal Society of Chemistry. (B) CeMIC microfluidic *C. elegans* sorting device with schematically illustrated peripheral equipment. Worms are measured for electrical impedance while flowing through the linear microchannel, and their developmental stage is identified on the basis of the impedance signal. Reproduced with permission from [110]. Copyright 2018 Elsevier. (C) Pressure-based microfluidic *C. elegans* sorting devices. (i) Microfluidic worm-sorter layout with control channels and valves (gray) that direct the flow of worms in the flow channels in different directions. Reproduced with permission from [73]. Copyright 2007 National Academy of Sciences, U.S.A. (ii) Microfluidic device consists of an array of geometrically optimized pillars. At low fluid pressure, the rigid pillars limit the movement of the larger worm through the device. However, at higher pressures, the pillars separate from the glass substrate, allowing the movement of larger worms. Reproduced with permission from [51]. Copyright 2014 Royal Society of Chemistry. (D) Continuous flow-based microfluidic *C. elegans* sorting device. The worms are encapsulated in droplets of M9 buffer solution surrounded by biocompatible oil. The tightly packed droplets flow continuously through the linear channel at a precisely defined rate. Worms are analyzed by image and then sorted from released droplets. Reproduced with permission from [111]. Copyright 2016 Elsevier. (E) Droplet-based microfluidic *C. elegans* sorting device consists of 2 functional regions: a T-junction droplet generator and a droplet trap array. Droplets are continuously generated at the T-junction and are immobilized in the droplet trap array. Reproduced with permission from [41]. Copyright 2014 Royal Society of Chemistry. (F) Fluorescence-assisted microfluidic *C. elegans* sorting device with optical fiber. When a worm passes through the detection region, it blocks part of the red light, which is detected by the first pair of fibers. The second pair of fibers excites and detects the green fluorescent protein (GFP) fluorescence of the worm if applicable, further differentiating the worm type. Reproduced with permission from [112]. Copyright 2014 Royal Society of Chemistry. UV, ultraviolet.

Stimulus response-based strategy depends on the behavioral responses of *C. elegans* to external stimuli. Electrotaxis is commonly applied in sorting among the physiological properties of *C. elegans* (Table 3). For microfluidic devices that utilize electrotaxis, it is relatively easy to create and control ac, dc, and dc pulses, and the application of electric fields has been developed for a long time that is very wide and can be controlled precisely [41]. Unlike the fluorescent sorting system described in Table 2 based on active imaging feedback, Rezai et al. [20] used local electric field traps coupled with the semi-continuous flow to sort larvae, separate mutants, and distinguish young and old adults passively. To improve the selectivity for different size animals, the local electric field is enhanced by narrowing the channel width (Fig. 5Ai). Similarly, Han et al. [22] applied an electric field to a hexagonally arranged microstructure optimized for worm size to achieve directional movement and maximum motility (Fig. 5Aii). Microfluidic impedance cytometry for cell-free detection is also utilized to detect large organisms like *C. elegans*. Zhu et al. [110] developed the CeMIC device to sort worms in the microchannel into different exits by measuring the electrical impedance related to the developmental stage (Fig. 5B). Pressure-based strategy is generally accomplished by controlling multiple valve openings and closings to adjust pressure for chamber cleaning, worm capture, release, and waste flushing. Adopting this strategy, Rohde et al. [73] designed a microfluidic worm sorter with control channels and valves that direct the flow of worms in the flow channels in different directions (Fig. 5Ci). Ai et al. [51] also developed a microfluidic device consisting of an array of geometrically optimized pillars. The pillars are controlled by the different fluid pressures (low or high) to limit or allow the movement of different sizes of worms (Fig. 5Cii).

Continuous flow-based and droplet-based strategies can be applied individually or combined for space fixation and restriction in microfluidic *C. elegans* sorting. Continuous flow indicates a constant flow of liquid. Yan et al. [111] designed a microfluidic device to segment a 2-layer PDMS that allows storing many droplets encapsulated with *C. elegans* and real-time analysis by image capture. A continuous flow drove these droplets through the linear channel, and the sorted worms were released from the droplets (Fig. 5D). In contrast, droplet represents the random movement of worms by using water (oil) to generate small chambers. Droplet-encapsulated individual worms can be continuously sorted and collected by specific structures [41]. For example, Shi et al. [90] developed a microfluidic *C. elegans* sorting device consisting of a T-junction droplet generator for generating droplets continuously and a droplet trap array for immobilizing (Fig. 5E). Aubry et al. [32] also achieved high-resolution imaging and sorting by encapsulating early *C. elegans* in thermosensitive hydrogel droplets.

The fluorescence-assisted strategy usually works on the basis of optical signal differences. Most microfluidic *C. elegans* sorting devices with this strategy perform a sequential "fixation-imaging-sorting" process. A few on-chip devices also apply a nonfixation approach, which increases sorting throughput to avoid potential pressure stimuli and flow imaging pauses. For instance, Yan et al. [112] developed a system to determine genotypes on the basis of fluorescence by a fiber optic detection system and switch laminar flow to change paths for sorting worms (Fig. 5F).

### Different Target *C. elegans* Sorting

**Fig. 6.**
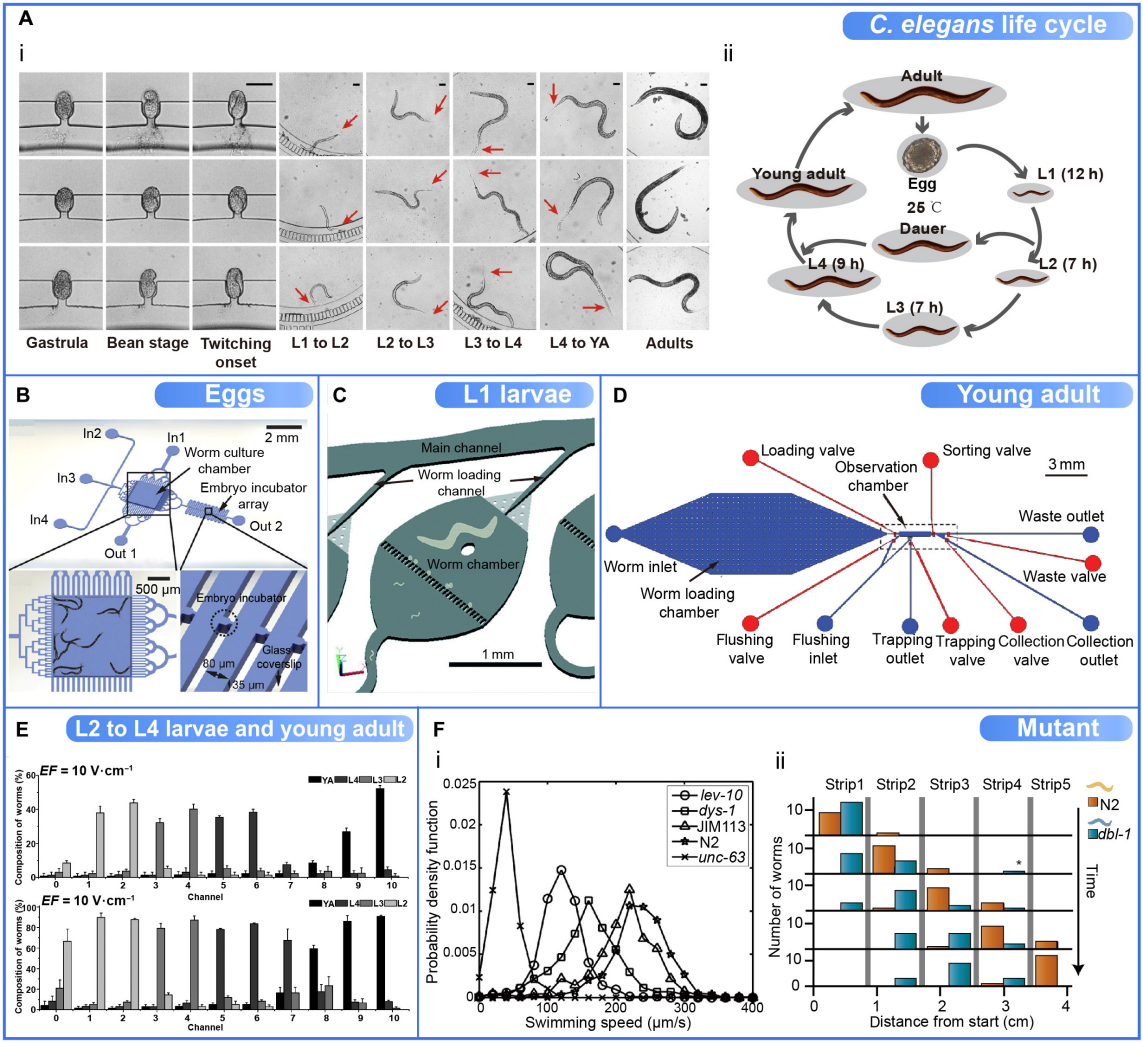
*C. elegans* populations sorted by microfluidic devices, and some sorting data (e.g., distribution data of different genotypes). (A) The life cycle of *C. elegans*. (i) Illustration of 3 worms at different life cycles. Reproduced with permission from [113]. Copyright 2018 Springer Nature. (ii) The life cycle of *C. elegans*. (B) The central part of the microfluidic chip consists mainly of a worm culture chamber, an embryo incubator array, 4 inlets (In 1 to In 4), and 2 outlets (Out 1 and Out 2). The embryo incubator array is specifically designed for capturing *C. elegans* embryos and their high-resolution imaging through glass coverslips. Reproduced with permission from [38]. Copyright 2015 Springer Nature. (C) Single chamber with a filter. The filter channel is 20 μm wide and allows only L1 worms to pass through, leaving unhatched eggs, unfertilized oocytes, and the mother retained inside the chamber. Reproduced with permission from [118]. Copyright 2015 Royal Society of Chemistry. (D) The worm loading chamber is supported by micropillar arrays for worm storage. After pressurization, only the larger worms (L4, young adult, and adult worms) remained in the chamber. The channel height of the top flow layer is 45 μm (approximately the average diameter of young adult worms), allowing for more accurate measurement and sorting of young adults. Reproduced with permission from [61]. Copyright 2019 IEEE. (E) Distribution and composition of 4 stages of *C. elegans* (L2 to L4 and young adult worms) in the deflected channels of the microfluidic device. Four stages of *C. elegans* mixed in equal proportions were sorted simultaneously under an electric field of 10 V·cm^−1^. Reproduced with permission from [55]. Copyright 2015 Royal Society of Chemistry. (F) Mutants were sorted from the wild type using microfluidic devices. (i) Abnormal mutation of the maximum swimming speeds of animals of the genotypes. The probability density functions of the maximum swimming speeds of animals of the genotypes: wild-type (N2, stars), wild-type expressing fluorescent protein (JIM113, triangles), *lev-10* (circles), *dys-1* (squares), and *unc-63* (crosses). Reproduced with permission from [122]. Copyright 2015 Royal Society of Chemistry. (ii) Neurodegenerative disease research of mutant. The numbers of wild-type worms (orange) and *dbl-1* mutant worms (blue) are shown as a function of time and space. Wild-type worms were sorted from the initial mix. The final strip contains only wild-type worms, while the second and third strips contain only *dbl-1* mutants. Reproduced with permission from [24]. Copyright 2011 PLOS ONE.

According to the subject demands and the equipment design, the target *C. elegans* that needs to be sorted by microfluidic devices is different. Sorting out and collecting many specific worms rapidly and accurately can accelerate studies on gene expression, neurodegenerative diseases, and drug discoveries, among others [9–12]. Table 5 provides an overview of *C. elegans* populations sorted by microfluidic devices. Worms in different developmental stages are one of the most common sorting targets. Figure 6Ai shows the whole life cycle of the worms on the worm array within the progeny culture chamber matrix, with arrows pointing to the detachment of the molts from the worm [113]. The developmental stages of *C. elegans* include the egg stage (embryo), 4 larval stages (L1 to L4), and the adult stage (Fig. 6Aii). The embryo develops entirely into an adult worm in approximately 3 to 4 d, with an average life span of 2 to 3 wk. Moult is the end of each larval stage to separate the developmental stages. Studies have shown that *C. elegans* is characterized by body size, morphology, and behavior at various stages [114,115].

**Table 5.** An overview of *C. elegans* populations sorted by microfluidic devices.

Target sorting population(s)	Capabilities	Reference(s)
Eggs (embryos)	Embryonic gene expression and morphogenesis	[38,46,49,93,113,116,117,162]
L1 larvae	Late embryonic development and other studies of developmental processes	[32,118]
L3 to L4 larvae	Genetics research with higher sample purity, test of size effect on the sorting effectiveness	[120]
L2 to L4 larvae	Simultaneous synchronization of massive different stages of worms for relevant studies	[55]
L1 to L4 larvae	Long-term and continuous larval phenotype observation and population acquisition	[46,121]
Adults of different ages	Neurodegenerative disease and aging research	[24,54,55,61]
All stages including adults	Biochemistry, disease, genetics, and more	[20–22,47–49,51,75,110]
Mutants from wild type	Mutation studies and drug discovery	[20,24,48,49,55,74,112,122,134,135]

**Table 6.** Summary of various sorting criteria on which microfluidic devices based.

Sorting criteria	Principle	Reference(s)
Size-based	Different developmental stages of worms are represented by a specific size range	[21,22,36,46–49,54,55,61,93,110,116,123,131,163–165]
Locomotion-based	Worms are sorted by differences in self-propelled movement (e.g., swimming frequency, speed) from one place to another.	[24,122,124,131]
Age-based	Determining age-related size phenotypes of larvae to sort different developmental stages	[20,54]
Fluorescent intensity-based	Phenotypic characterization of worms based on fluorescence intensity was imaged and sorted.	[50,120]
Mutation-based	Mutants lacking a certain physiological response (e.g., electrotactic response) due to a genetic defect are isolated.	[20,24,8,49,55,74,112,122,134,135]

By hatching sorted embryos, it is possible to obtain a large number of accurately age-matched synchronized populations for the studies of embryonic gene expression and postembryonic morphogenesis [46,113,116,117]. To sort the *C. elegans* embryos, Atakan et al. [46] developed a microarray using purely passive hydrodynamics capable of gently sorting and immobilizing massive embryos, which can benefit the study of morphogenesis and mitochondrial biogenesis using *C. elegans* embryos. Cornaglia et al. [38] also designed a microfluidic device using purely passive fluid dynamics to obtain synchronized embryo populations left in special serpentine channels and high-resolution imaging in an incubator microarray (Fig. 6B).

The first larval stage (L1) is challenging to manipulate and tends to clog most microfluidic microarray devices, and the L1 is essential for many biological studies, such as late embryonic developmental processes [32,118]. Li et al. [118] developed a microfluidic device containing single chambers with filters. The width of the filter channel only allowed the L1 larvae to pass through and thereby be sorted, while the remaining stages of *C. elegans*, e.g., unhatched eggs, were retained in the chamber (Fig. 6C).

The growth of worms can be affected by the cultivation environment. For example, under environmental stress, L2 larvae can develop into the dauer stage [3,13,119]. Because of this abnormal development, L2 is often sorted for environmental influences studies. Because L3 and L4 have larger body sizes (length and diameter) and are easily identified under a dissecting microscope, their synchronized populations were used to test the effect of size on the sorting effectiveness [120]. Different stages of *C. elegans* exhibit behavioral differences in sorting young and old adults for aging and neurodegenerative disease studies. Dong et al. [61] designed a microfluidic device with a worm-loading chamber supported by micropillar arrays. After pressurization, only the larger worms (L4, young adult, and adult worms) remained in the chamber. The channel height of the top flow layer is 45 μm (approximately the average diameter of young adult worms), allowing for more accurate measurement and sorting of young adults (Fig. 6D). Sorting for the whole larval stage (L1 to L4) permits long-term larval phenotypic shape observation [46,121]. An increasing number of devices can study all stages including adults, facilitating biochemistry, disease, genetics, and more. Compared to the engineers' attention to design ideas of microfluidic sorting devices, biologists are more interested in the characteristics and values of different target *C. elegans* populations. Sorting data of *C. elegans* (e.g., distribution data of different genotypes) can provide biologists with a diverse perspective in more biological studies. A microfluidic device based on deflecting electrotaxis was developed by Wang et al. [55]. As shown in Fig. 6E, 4 stages of *C. elegans* (L2 to L4 and young adult worms) populations showed different distribution and composition in different channels under an electric field of 10 V·cm^−1^.

Genotype affects the motility of animals. Yuan et al. [122] used a motion-based microfluidic device to sort rare mutants from the wild type, and forward genetic screening was performed. As shown in Fig. 6Fi, the wild type (N2) and various mutants (*lev-10*, *dys-1*, JIM113, and *unc-63*) exhibited different maximum swimming speeds. Manière et al. [24] designed a simple device using electrotaxis to enable *C. elegans* with different locomotor phenotypes to self-classify in time and space. Wild-type worms were sorted from the initial mix. Figure 6Fii shows that the final strip contains only wild-type worms, while the second and third strips contain only *dbl-1* mutants. A group of *C. elegans* with comparable motor adaptations can be isolated to study neurodegenerative diseases, improving the high-throughput search for therapeutic biomolecules.

### Microfluidic Devices Based on Various Sorting Criteria

Rapid growth and reproduction of worms can lead to population complexity after a short period. For biologists, the most interesting perspective could be the sorting criteria, i.e., what type of worms can be sorted out by microfluidic devices. Several phenotypic characteristics of worms can be used as sorting criteria for different microfluidic device designs, such as size-based and locomotion-based.

**Fig. 7.**
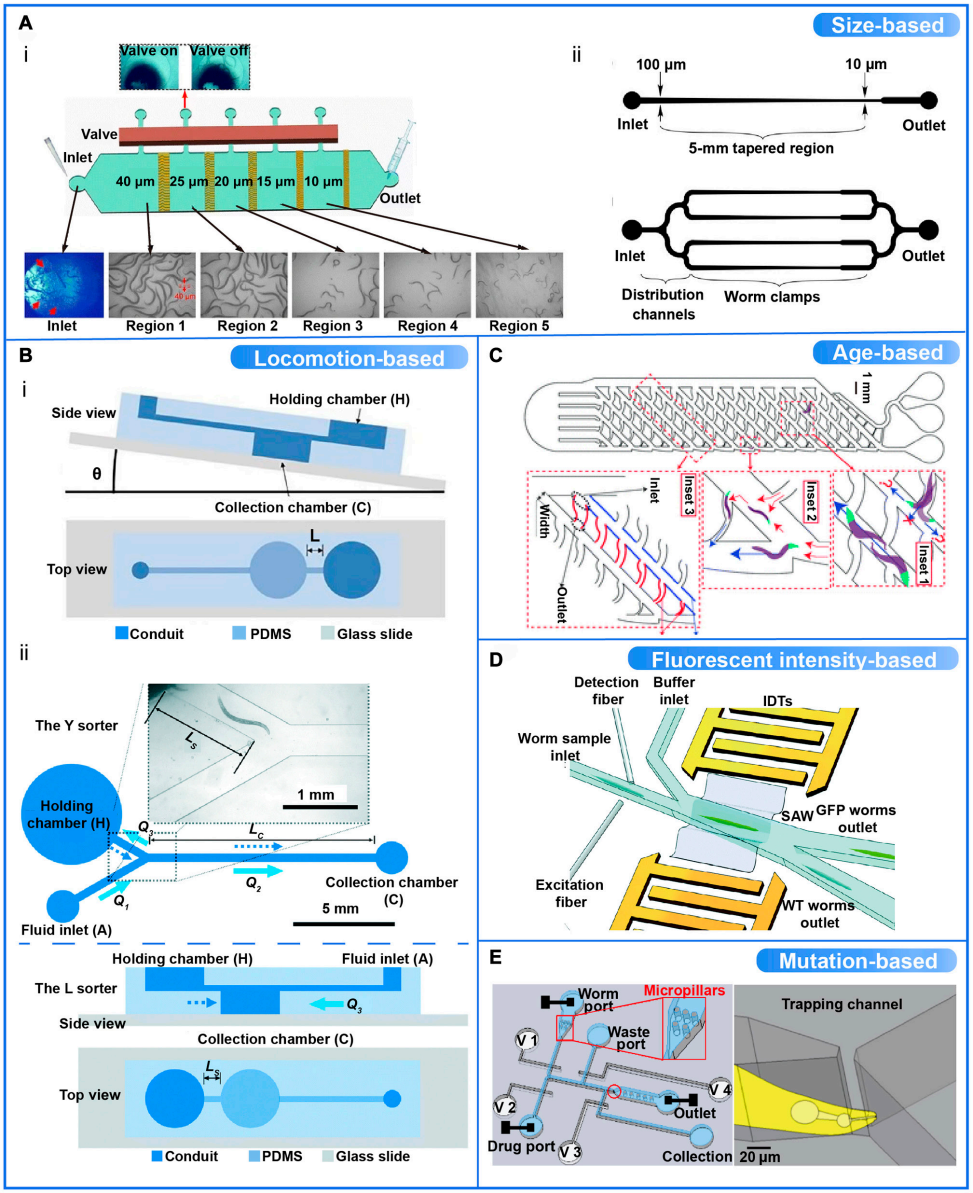
Various sorting criteria on which microfluidic devices were based. (A) Size-based microfluidic sorting devices. (i) Size-based microfluidic sorting device with diode arrays. Worms of different sizes from the original mixture are efficiently sorted in different regions (Regions 1 to 5). Reproduced with permission from [47]. Copyright 2017 Springer Nature. (ii) Size-based microfluidic arrays designed for single or multiple worms. Microchannel widths taper to accommodate worms of different sizes. Reproduced with permission from [36]. Copyright 2007 Royal Society of Chemistry. (B) Locomotion-based microfluidic sorting devices. (i) Gravity-assisted microfluidic *C. elegans* sorting device with an inclined conduit. Worms entering the device are deposited at the bottom of the collection chamber and sorted by differences in the propulsive force of different worms along an inclined plane. Reproduced with permission from [124]. Copyright 2016 Royal Society. (ii) Locomotion-based Y sorter and L sorter with conduits and syringe pumps selectively sort worms with propulsive power above the preset threshold by controlling the flow rate. Reproduced with permission from [122]. Copyright 2018 Royal Society of Chemistry. (C) The “smart mazes” with a network of intercommunicating channels allow the adults to move easily toward the desired outlet and to sort the larvae of different ages. Reproduced with permission from [54]. Copyright 2011 Royal Society of Chemistry. (D) The fluorescent intensity-based acoustofluidic worm sorting device with a microchannel, a pair of optical fibers, and a pair of interdigital transducers. Each *C. elegans* is genotypically differentiated after the alignment of the 2 optical fibers, generating the corresponding optical signal. Reproduced with permission from [120]. Copyright 2020 Royal Society of Chemistry. (E) Mutation-based 2-layer NeuroChip for processing worms and delivery of drugs and magnified trapping channel (red circled). Reproduced with permission from [74]. Copyright 2013 PLOS ONE. WT, wild type; IDT, interdigital transducer; SAW, surface acoustic waves.

Because of the size-dependent characteristics of *C. elegans* and their easily observable body size, size-based sorting is one of the most common sorting criteria. Microfluidic devices based on such criteria typically limit worm space by employing geometric restriction [36,123]; thereby only those meeting the minimum passage size can pass. Yang et al. [47] developed a microfluidic sorting device with diode arrays. By limiting the width of each diode array, worms of different sizes from the original mixture are efficiently sorted in different regions (Fig. 7Ai). Hulme et al. [36] also designed a microfluidic array with tapered microchannel widths that restrict worms of different sizes to sort them (Fig. 7Aii).

Because of differences in genotype, gait, and disease state, *C. elegans* may differ in its locomotion in flat or inclined planes. Therefore, Yuan et al. [124] developed a gravity-assisted microfluidic *C. elegans* sorting device with an inclined conduit. Worms entering the device are deposited at the bottom of the collection chamber and sorted by differences in the propulsive force of different worms along an inclined plane (Fig. 7Bi). Similarly, they also designed a variant device including 2 types, Y sorter and L sorter, using syringe pumps designed to control the flow rate (Fig. 7Bii), which can sort worms according to their swimming ability in the liquid [122]. *C. elegans* with propulsive forces above the preset threshold (upstream movement) are separated.

Aging and genetic analyses require large age-synchronous worm populations for biological studies. Hence, age-based criteria about phenotypic differences in sorting are used for microfluidic devices based on the worm's propensity for strategic movement in stimuli response, such as chemicals or food, and adult stronger swimming performance in specific environments [125,126]. Casadevall i Solvas et al. [54] developed a “smart mazes” microfluidic device with a network of intercommunicating channels that allows the adults to move easily toward the desired outlet and to sort the larvae of different ages (Fig. 7C).

Fluorescent intensity-based sorting devices use the expression of fluorescent proteins in a continuous flow to identify and separate target worms. Zhang et al. [120] proposed an integrated acoustofluidic device that uses planar optical fibers and surface acoustic waves as detection and sorting units, respectively, to move the target worms toward the desired exit without contact (Fig. 7D).

Microfluidic parameters are typically designed on the basis that all size-based sorting assays are carried out using wild-type worms. However, genetic mutations can cause abnormal worm body sizes, and a few microfluidic devices will fail to work accurately. Unlike the heterogeneous filters designed in size-based sorting, the mutation-based microfluidic sorting devices operate by utilizing genes that result in different physiological phenotypes to sort multiple genotypes of *C. elegans*. For example, He et al. [74] developed a microfluidic electrophysiological device called NeuroChip, which is based on the electrophysiological phenotype of the worm pharynx. This device combines microfluidic- and integrated-electrode recording with pneumatic microvalves for *C. elegans* collection and releases nondestructively (Fig. 7E). It provides new tools for neurogenetics, drug discovery, and neurotoxicology studies.

## Future Prospects for Microfluidic *C. elegans* Sorting Devices

As discussed in the sections above, microfluidic devices offer various advantages, such as cost-effective, convenient, and user-friendly solutions for *C. elegans* sorting compared to the conventional manual sorting and COPAS system. First, the microfluidic devices use active methods with microvalves and micropumps to control flow and pressure, or the passive methods with microstructures to alter the fluid flow containing the worms. Second, the sorting strategies for microfluidic devices are more detailed and include stimulus response-based, continuous flow-based, and droplet-based, among others. These strategies are created around the ability of the device itself to manipulate the liquid and the worm properties. Third, depending on design expectations, device fabrication, research direction, and sorting principles, microfluidic devices can sort a wide range of target worm populations, for example, eggs, L3 to L4, and young adults. Fourth, microfluidic devices developed sorting criteria based on the potential phenotypic differences in each worm, such as mutation-based (phenotypic abnormalities) and locomotion-based. In addition, aspiration channels and imaging microchannels, for instance, can provide gentle fixation and high-resolution imaging, enhancing the observation and analysis of subtle phenotypes. Through integrated algorithms or other computer aids, microfluidic devices can be effectively assisted in data collection, processing, and analysis with reduced manual intervention and reproducibility.

Though powerful in sorting, microfluidic devices have some limitations on fabrication and operation. The fabrication of microfluidic devices for PDMS substrates typically needs ultraviolet lithography, which requires expensive equipment and spaces such as spin coaters and clean rooms to prevent substrate damage by solid particles in the air. Thus, optimizing the laboratory operating environment, including fluid flow and temperature, is required.

Research on *C. elegans* has been conducted for over half a century, and more laboratories are embracing and developing microfluidics for sorting, chemical screening, and genetic detection. The increased precision of additive manufacturing and other fabrication techniques has great potential for more laboratories that lack the conditions to use this technology. The marketing of next-generation microfluidic commercial devices will be mutually reinforcing with laboratory research and development.

Many devices available for *C. elegans* sorting have been demonstrated here. Still, more microfluidic devices, such as defective neuronal screening, will be developed for more extensive scale and higher-throughput specialized sorting. Most current microfluidic platforms designed specifically for sorting use serial studies of worms recovering their progeny and remain challenging for long-term studies, such as forward genetic screening. Parallelization and long-term tracking studies are some future directions for microfluidic sorting platforms. The abundant phenotypic and physiological properties of worms are the basis for more possible sorting criteria or sorting strategies of microfluidic devices. By adding additional sequencing channels and the number of inlets, it will be possible to increase the throughput and prescreening capacity of the device. In conclusion, developing microfluidic devices or platforms for nematode sorting should be guided by practical applications and experimental needs, exploring more possibilities based on the worms' advantages and enhancing the devices' robustness, precision, and efficiency. Future development cannot be limited to the mere patchwork of multiple techniques or the deliberate pursuit of novel but inefficient and expensive devices.

## Conclusions

Over the decades, *C. elegans* has been a model organism with significant potential for human diseases and genetics research. Compared to the tedious and inefficient conventional manual methods and the commercial sorter COPAS, which is unaffordable for most laboratories, the development of lab-on-a-chip (microfluidics) technology has efficiently and cost-effectively provided large populations of synchronized worms through innovations in device structures, sorting strategies, and automation algorithms. Most previous reviews have focused on the development of microfluidic devices but lacked the summaries and discussions of the biological research demands of *C. elegans*.

We comprehensively reviewed the up-to-date microfluidic-assisted *C. elegans* sorting developments from several angles. First, we summarized the advantages and limitations of microfluidics-assisted *C. elegans* sorting technology (e.g., functionality, manipulation, and throughput) to facilitate a clearer understanding for researchers from different backgrounds. Second, we classified existing devices in terms of active or passive sorting, physiological characteristics, and sorting strategies to provide engineers with more new design ideas for microfluidic devices. Third, we also classified existing devices in terms of physiological characteristics, target worm populations, and sorting criteria to provide biologists with a more diverse perspective in drug discovery, neurogenetic research, and other biological studies. We expect that this comprehensive review can provide an effective and well-organized documentary for researchers in this cross-section and facilitate the development of this multidisciplinary area. While there are some drawbacks in the fabrication and operation of microfluidic devices, the future of serving more laboratories is promising. We also expect the new microfluidic devices will contribute to optimizing *C. elegans* sorting for faster implementation into practical applications.

## Data Availability

The data that support the findings of this study are available from the corresponding author upon reasonable request.
